# Oral streptococcal infective endocarditis among individuals at high risk following dental treatment: a nested case-crossover and case-control study

**DOI:** 10.1016/j.eclinm.2023.102184

**Published:** 2023-08-30

**Authors:** Niko Vähäsarja, Bodil Lund, Anders Ternhag, Bengt Götrick, Lars Olaison, Margareta Hultin, Carina Krüger Weiner, Aron Naimi-Akbar

**Affiliations:** a Division of Oral Diagnostics and Rehabilitation, Department of Dental Medicine, Karolinska Institutet, Stockholm, Sweden; b Department of Oral and Maxillofacial Surgery, Eastmaninstitutet, Folktandvården Stockholms Län AB, Folktandvården Eastmaninstitutet, Stockholm, Sweden; c Medical Unit of Plastic Surgery and Oral and Maxillofacial Surgery, Department for Oral and Maxillofacial Surgery and Jaw Orthopedics, Karolinska University Hospital, Stockholm, Sweden; d Department of Oral Diagnostics Faculty of Odontology, Malmö University, Malmö 205 06, Sweden; e Department of Infectious Diseases, Institute of Biomedicine, Sahlgrenska University Hospital, Blå Stråket 5, Göteborg 413 45, Sweden; f Division of Infectious Diseases, Department of Medicine Solna, Karolinska Institutet, Stockholm 171 77, Sweden; g Health Technology Assessment-Odontology (HTA-O), Faculty of Odontology, Malmö University, Malmö 205 06, Sweden

**Keywords:** Prophylactic antibiotics, Dentistry, Infective endocarditis, Viridans group streptococci

## Abstract

**Background:**

It is not clear whether Viridans Group Streptococcal Infective Endocarditis (VGS-IE) among individuals at high risk is more frequent following bacteraemia caused by invasive dental procedures (IDPs) than after daily bacteraemia caused by chewing and tooth brushing. The aim of this nested study was to assess if VGS-IE was temporally associated with IDPs in a national cohort of individuals at high risk.

**Methods:**

This nested case-control and case-crossover study was based on a Swedish national cohort study of 76,762 individuals at high risk of IE due to complex congenital heart disease, prosthetic heart valve or previous IE. Participants were living in Sweden between July 1st, 2008 and January 1st, 2018. The frequency of IDPs during the 3 months before VGS-IE was calculated and compared to controls (sampled 1:10). A case-crossover study was conducted to account for residual confounders. Participants were identified using the national patient register, and IDPs were identified using the national dental health register.

**Findings:**

98,247 IDPs were carried out in the cohort during the study period: 624 occasions of oral surgery, 44,190 extractions and 53,433 sessions of subgingival scaling. The study could not confirm that IDPs were more common among cases (4.6%) than controls (4.1%), OR = 1.22 [95% Confidence Interval (CI) 0.64–2.3], or during case- (3.3%) than reference periods (3.8%), OR = 0.89 [95% CI: 0.68–1.17]. Restricting the analysis to the period when cessation of antibiotic prophylaxis for the prevention of IE in Swedish dentistry was recommended, from the 1st of October 2012 to the 1st of January 2018, did not alter the results of the case-control study: OR 0.64, 95% CI: 0.20–2.09, or the case-crossover study: OR 0.58, 95% CI: 0.15–2.19.

**Interpretation:**

The study could not confirm that VGS-IE is associated with IDPs among individuals at high risk. A study with larger sample size could clarify whether there is a lack of association. The finding of a small (<5%) proportion of cases temporally associated with IDPs is similar to that of the previous large-scale study on IDPs and VGS-IE.

**Funding:**

Funding was provided by the Board of doctoral education at 10.13039/501100004047Karolinska Institutet, the 10.13039/501100010686Public Health Agency of Sweden, Folktandvården Stockholm AB, Steering Group for Collaborative Odontological Research at 10.13039/501100004047Karolinska Institutet and Stockholm City County, and the 10.13039/501100005300Swedish Dental Association.

**Research in context**

**Evidence before this study**

We searched PubMed on January 8, 2023, for articles published in English using the search terms “infective endocarditis”, “viridans streptococci”, “dental procedures” with no date restrictions. Large scale evidence on an association between invasive dental procedures (IDPs) and viridans group streptococcal infective endocarditis (VGS-IE) among individuals at high risk is limited to one cohort- and case crossover study from France that showed ambiguous results. 5% of cases were temporally associated with IDPs. Two recent studies on IE among individuals at high risk in USA show an association with tooth extractions and oral surgical procedures, and an inverse association with scaling procedures, although a lack of microbiological data render the results difficult to interpret.

**Added value of this study**

This is the first Swedish nationwide study to evaluate the association between IDPs and VGS-IE among individuals at high risk. Our nationwide study identified 76,762 adults who had undergone 98,247 IDPs over almost ten years. The study could not confirm any association between IDPs and VGS-IE. The results confirmed a minor proportion of VGS-IE <5% were temporally associated with IDPs. Larger studies on IDPs and VGS-IE among individuals at high risk could help determine whether there is an association.

**Implications of all the available evidence**

Our findings suggested that most cases of VGS-IE among individuals at high risk do not occur during the three months following IDPs. The association between IDPs and IE found in two recent studies implies a strong association between IDPs and VGS-IE, however this is yet to be confirmed in studies with access to microbiological data.

## Introduction

It is not clear whether the risk if VGS-IE (Viridans Group Streptococcal Infective Endocarditis) among individuals at high risk is elevated following bacteraemia caused by invasive dental procedures (IDPs) compared to daily bacteraemia. If such an association does not exist, the use of prophylactic antibiotics (AP) in dentistry for the prevention of VGS-IE is not clinically meaningful.

Individuals with prior IE, prosthetic heart valve, or complex congenital heart disease (CHD), are considered at high risk, because incidence rates of IE among these groups are increased compared to the general population.^1^, ^2^, ^3^, ^4^, ^5^ 25–30% of IE-cases are caused by VGS commonly found in the oral cavity,^5^, ^6^, ^7^ and may be associated with chewing, brushing, or dental procedures.^8^ According to European and American guidelines, individuals at high risk should receive AP prior to IDPs (dental extractions, the removal of dental calculus, and oral surgical procedures) to reduce the risk of VGS-IE.^8^^,^^9^ Expert committees revising the guidelines acknowledge that it is unclear whether AP prevents VGS-IE and that VGS-IE is more likely the result of daily bacteraemia caused by chewing and brushing of teeth, than by occasional dental procedures.^8^^,^^9^ Recent studies on national data from England and Sweden (where the prophylaxis is no longer routinely recommended since 2008 and 2012, respectively), detected no increase in VGS-IE associated with the guideline change.^5^^,^^6^ In England an addendum in 2016 stated that the prophylaxis is not recommended routinely, although amoxicillin prescriptions did not increase among dentists after this change.^6^ In Sweden an addendum published in March 2016, specifies that AP is not routinely recommended. The addendum states that AP may be considered if advised by the patient’s physician. In such cases, the physician is responsible for notifying the dentist.^10^ No increase in amoxicillin prescriptions among dentists was detected after the publication of the addendum.

If there is an association between IDPs and VGS-IE among individuals at high risk it would be easier to detect in Sweden or England, where it may not be masked by the use of AP.^7^ Thornhill et al. attempted to study the association in England, although as the data showed a significant decrease in IDPs during the 3 months prior to IE, the authors attributed this to selective loss of data.^7^

The aim of this nested study was to evaluate whether VGS-IE among the individuals at high risk was temporally associated with IDPs.

## Methods

### Study design

This nested case-control and case-crossover study was based on a nationwide cohort study of individuals at high risk of IE (Table 1) described in detail elsewhere.^5^ The original cohort consisted of all adult individuals (≥18 years of age) living in Sweden between January 2008 and January 2018, with an ICD (International Classification of Diseases) code and/or surgical procedure code indicating high risk of IE in the Swedish National Patient Register (NPR), or Medical Birth Register (MBR). The study is reported according to STROBE (statement for reporting case-control studies) guidelines.^11^

**Table 1:** Characteristics of study participants.

	Cases	Controls	Case-crossover
N	240	2373	213
Age category (%)			
<30	11 (5)	108 (5)	9 (4)
30–39	19 (8)	178 (8)	16 (8)
40–49	19 (8)	180 (8)	15 (7)
50–59	34 (14)	339 (14)	29 (14)
60–69	42 (18)	420 (18)	39 (18)
70–79	67 (28)	670 (28)	61 (29)
80+	48 (20)	478 (20)	44 (21)
Sex (%)			
Female	68 (28)	664 (28)	60 (28)
Education (%)			
Missing/not completed compulsory education	0	0	0
Compulsory/upper secondary	88 (37)	855 (36)	78 (37)
Post secondary	106 (44)	1060 (45)	96 (45)
Post graduate education	46 (19)	458 (19)	39 (18)
Comorbidities			
Diabetes	45 (19)	376 (16)	43 (20)
Intravenous substance abuse	15 (6)	78 (3)	14 (7)
ICD/pacemaker	61 (25)	641 (27)	53 (25)
IV catheter/hemodialysis	8 (3)	53 (2)	8 (4)

Distribution of age, sex, educational attainment and comorbidities among the cases and controls included in the case-control and case-crossover study.

**Fig. 1:**
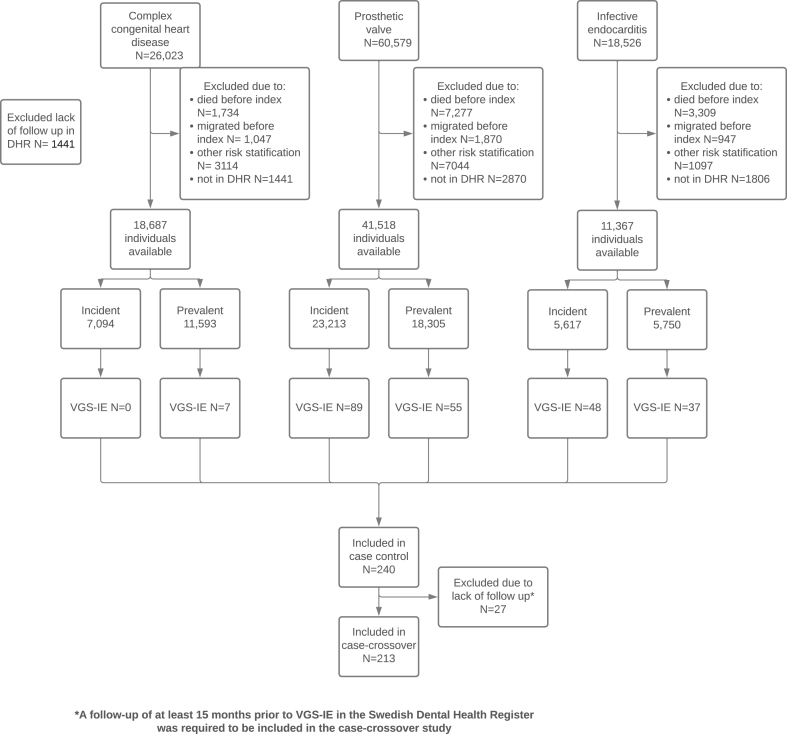
**Flow chart—inclusion of participants.** Legend: Flow chart describing the inclusion of participants in the case control- and case crossover study. VGS-IE: Viridans Group Streptococcal Infective Endocarditis; Index: the point in time at which the participant started contributing with time at risk in the original cohort study on which this study is based; DHR: the Swedish Dental Health Register; Incident: participants included due to registered risk factors between 1st of January 2008 and 1st of January 2018; Prevalent: participants included due to registered risk factors between 1st of January 1964 and 1st of January 2008.

Individuals were considered at high risk of IE if they had a recorded primary or secondary ICD- and/or surgical procedure code indicating CHD, heart valve prosthesis or previous IE (Fig. 1) in the NPR and MBR since 1964 and 1973, respectively.^5^

Although the original cohort contained individuals ≥18 years of age, and follow up started on the 1st of January 2008, the current study included individuals ≥24 years of age and follow up started on the 1st of July 2008. This was because data on the exposure IDPs in the Swedish Dental Health Register (DHR) is limited to these individuals.^10^ The current case-control study was able to include three additional cases of VGS-IE compared to the original cohort study (240 cases instead of 237). This was due to including all first-time cases of VGS-IE available, whereas in the previous study, follow up time was limited to two years among incident individuals at high risk to minimise risk of bias.^5^ The outcome, cases of VGS-IE, were identified using the national Swedish quality Register for Infective Endocarditis (SRIE), and fulfilled the criteria for definite or possible endocarditis according to the modified Duke criteria.^12^ VGS-IE was considered likely to be of oral origin and thus of relevance.

Using the DHR, data was gathered on the occurrence of IDPs during the 90 days prior to hospitalisation due to VGS-IE. The 90-day period was chosen to ensure comparability to previous studies.^4^^,^^7^^,^^13^, ^14^, ^15^, ^16^, ^17^, ^18^ Dental extractions, subgingival dental scaling (removal of dental calculus), and oral surgical procedures were considered IDPs (Table 4).

**Table 2:** Exposure to dental procedures among 213 participants with oral streptococcal infective endocarditis included in the case-crossover study, 2008–2018.

Period		N (%)
All procedures		
Case period (N = 213)		
Invasive dental procedure		7 (3.3)
Control periods (n = 426)		
Invasive dental procedure		16 (3.8)
OR	0.86	95% CI: 0.34–2.18
Extractions		
Case period (N = 213)		
Extractions		4 (1.9)
Control periods (n = 426)		
Extractions		7 (1.6)
OR	1.14	95% CI: 0.33–3.90
Scaling		
Case period (N = 213)		
Scaling		4 (1.9)
Control periods (n = 426)		
Scaling		9 (2.1)
OR	0.87	95% CI: 0.24–3.11

Number, proportion and types of invasive dental procedures temporally associated with oral streptococcal infective endocarditis among the 213 cases included in the case-crossover study.

**Table 3:** Exposure to dental procedures among 240 participants with oral streptococcal infective endocarditis included in the case-control study, 2008–2018.

		N (%)
All procedures		
Cases (n = 240)		
Invasive dental procedure	11 (4.6)	
Controls (n = 2373)		
Invasive dental procedure	98 (4.1)	
OR	1.22	95% CI: 0.64–2.3
Extractions		
Cases (n = 240)		
Extraction		6 (2.5)
Controls (n = 2373)		
Extraction		31 (1.31)
OR	1.87	95% CI: 0.78–4.50
Scaling		
Cases (N = 240)		
Scaling		5 (2.1)
Controls (n = 2373)		
Scaling		48 (2.0)
OR	1.04	95% CI: 0.41–2.67

OR: odds ratio; CI: confidence interval.

Number, proportion and types of invasive dental procedures temporally associated with oral streptococcal infective endocarditis among the 240 cases, and occurring during control periods among the 2373 controls included in the case-control study.

**Table 4:** Procedural codes used to identify invasive dental procedures in the dental health register.

Procedure	Codes
Tooth extraction	401–406
Dental scaling	342, 343
Oral surgery	405, 407–409, 429, 453, 454

Codes for identification of invasive dental procedures in the Swedish Dental Health Register.

### Case-control

Cases were defined as all individuals at high risk from the original cohort who developed VGS-IE according to the SRIE from July 1st, 2008 to December 31st 2018 (Fig. 1). Controls (1:10) who had not developed VGS-IE were sampled from the cohort by incidence density sampling, matching on risk factor, age, sex, and time span with risk factor. This resulted in a sample of 2373 controls matched to the 240 individuals who developed VGS-IE (Table 1). Occurrence of IDPs (Table 4) during the 90 days prior to hospitalisation due to VGS-IE was compared to controls. Odds Ratios (OR:s) were calculated using conditional logistic regression. Additional analyses were conducted across the three IDPs to account for potential differences in risk between procedures. Information of comorbidities prior to study onset was gathered from the NPR and MBR (Table 1).

### Case-crossover

**Fig. 2:**
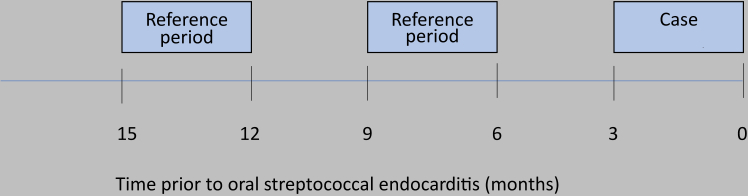
**Visual depiction of the case- and reference periods in the case-crossover study.** Legend: In the case-crossover study, the occurrence of invasive dental procedures during reference periods were compared to that of the case period, prior to oral streptococcal infective endocarditis.

A case-crossover study was carried out to account for selection bias of controls and residual confounders.^19^ The case-crossover design compares the frequency of exposure among case periods, with that of reference periods (Fig. 2). The study design has been used before in this field.^4^^,^^7^ Because the cases serve as their own controls, intraindividual confounders that do not vary over time are adjusted for per design.

The study population consisted of all individuals in the original cohort who developed VGS-IE and were theoretically covered by the DHR (≥24 years of age, living in Sweden) for at least 15 months prior to hospitalisation (Fig. 1). Occurrence of IDPs 90 days prior to hospitalisation, was compared to reference periods; month 7–9 and 13–15 preceding hospitalisation (Fig. 2). OR:s were calculated using conditional logistic regression. Additional analyses were conducted across the three different IDPs to account for potential differences in risk between procedures. Information of comorbidities (Table 1) prior to study onset was gathered from the NPR and MBR.

### Sources of data

#### Swedish National Register of Infective Endocarditis

The Swedish Society for Infectious Diseases established the SRIE in 1995. The register was used to identify cases of VGS-IE among the individuals at high risk. All 30 infectious disease departments in Sweden have participated in the registry since it was created and the coverage has been estimated to 88% of all cases of IE.^20^ These departments are responsible for providing care to patients with severe infections, and patients who require emergency surgery for infectious endocarditis (IE) are typically treated in these departments before and/or after the surgery. All cases are documented using standardised forms at the time of discharge and again after follow-up (average of 3 months after treatment). The form includes information about risk factors, the presence of prosthetic valves and other implantable cardiac devices, and the type of prosthetic valve. The cause of the infection is determined using methods such as blood cultures, cultures from valves during surgery, and 16S RNA sequencing of tissue samples from valves.

#### Total population register

Data on sex, age, educational attainment, and date of death was obtained from the total population register held by Statistics Sweden, the government agency responsible for developing, producing, and disseminating official statistics and other government statistics in Sweden. All study persons included in this study had complete data on these variables.

#### Dental health register

Data on IDPs previously considered cause for AP in Sweden (Table 4) was obtained from the Swedish DHR.

The DHR is a national database containing information on dental care services compensated by the Swedish state. Children and adults under the age of 24 receive free dental care publicly funded through taxes, and procedures are not included in the DHR. The DHR was established in 2008 and is administered by the Social Insurance Agency (SIA). All dental clinics are required to report procedural codes (Table 4) to the SIA to receive financial compensation from the national dental care subsidy, that covers all Swedish residents insured and covered by the Swedish social insurance system. The coverage is estimated to be nearly complete nationally.^10^ In Sweden, electronic dental record systems are connected to the SIA, and procedural data is transferred automatically prior to debit. The data is then transferred monthly from the SIA to the DHR held by the National Board of Health and Welfare, responsible for maintaining the national health data registers. The DHR is governed by legislation issued by the Swedish government and is used for a variety of purposes, including the evaluation and planning of health care services, monitoring public health, and conducting research. Electronic reporting is mandatory for dental health professions.^10^ Notably, dental procedures and surgery carried out in hospitals are not covered by the national dental care subsidy and are not reported to the DHR, but to the NPR. The cost of these procedures is included in the high-cost protection for other health and medical care. These are not included in the current study.

### Statistical analysis

OR:s were calculated using conditional logistic regression. The statistical analysis was carried out using Stata/IC 15 (StataCorp. 2017. Stata Statistical Software: Release 15. College Station, TX: StataCorp LLC). All study persons had complete register data on age, sex, educational attainment and comorbidities (Table 1). All cases of VGS-IE among the study persons available in the SRIE were included as outcome data.

### Ethics

This study was approved by the Regional Ethical Review Board, Karolinska Institutet, Stockholm, Sweden (ref: 2018/370-31) prior to onset of the study.

An ethical permit and approval for the study was also obtained from the register board of the SRIE, before the collection of data.

Informed consent was waived because the study does not pose any risk of harm to the participants; no identifying information is published as results are presented on group level only.

### Patient and public involvement

The patients and public were not involved in the preparation of the study design, dissemination of results, or evaluation of this study.

### Role of the funding source

All authors NV, BL, BG, MH, AT, LO and CKW had full access to all the data in the study and accept responsibility for the decision to submit for publication. The study sponsors had no input in the in study design, the collection, analysis, or interpretation of data, in the writing of the report, or in the decision to submit the paper for publication.

## Results

Between the 1st of July 2008 and the 1st of January 2018, 240 cases of VGS-IE occurred among the 76,762 individuals at high risk (Fig. 1). During the same period, 98,247 IDPs were carried out in the cohort; 624 occasions of oral surgery, 44,190 tooth extractions and 53,433 sessions of subgingival scaling (removal of dental calculus).

**Fig. 3:**
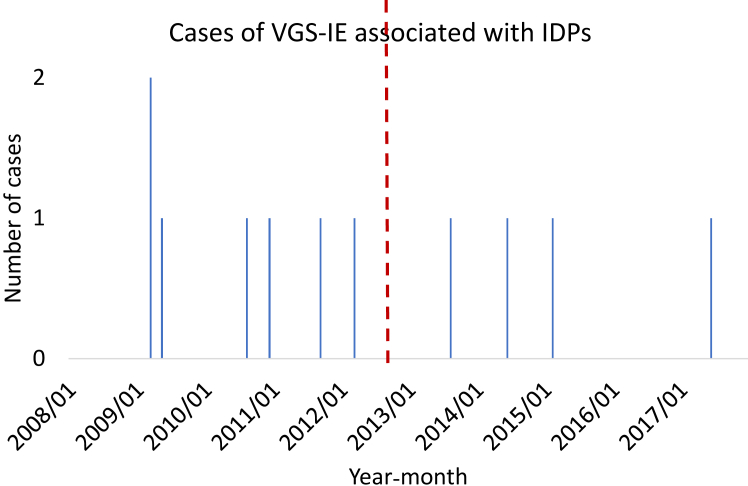
**Cases of oral streptococcal infective endocarditis temporally associated with invasive dental procedures.** Legend: The red dotted line represents the time of the recommended cessation of antibiotic prophylaxis in dentistry for the prevention of viridans streptococcal infective endocarditis (VGS-IE) in October 2012. Four out of the eleven invasive dental procedures temporally associated with VGS-IE occurred after the change.

The case-control study could not confirm that IDPs were more common among cases than controls (Table 3) OR = 1.22, 95% Confidence Interval (CI): 0.64–2.3. 11 (4.6%) cases and 98 (4.1%) controls underwent IDPs during the 3 months preceding VGS-IE. 4 out of the 11 cases temporally associated with IDPs occurred after October 2012, when cessation of AP for the prevention of IE in Swedish dentistry was recommended (Fig. 3).

The case-crossover study could not confirm that IDPs were more common during the 3-months prior to VGS-IE than during reference periods, OR = 0.86, 95% CI: 0.34–2.18 (Table 2). Stratification on procedure did not alter these results. No OR could be calculated for oral surgery because no such procedures were detected during case-or reference periods.

Restricting the analysis to the period when no AP for the prevention of IE was recommended, from the 1st of October 2012 to the 1st of January 2018 did not alter the results of the case-control study: OR 0.64, 95% CI: 0.20–2.09, or the case-crossover study: OR 0.58, 95% CI: 0.15–2.19.

Restricting it to the period from the 1st of October 2012 to the 1st of March 2016, the OR of the case-control study was 0.76, 95% CI: 0.18–3.28, and the OR of the case-crossover was 1.00, 95% CI: 0.18–5.45.

Restricting the analysis to the period when AP for the prevention of IE was recommended, in the 1st of July 2008 to the 1st of October 2012, yielded the following results for the case-control study: OR 1.80, 95% CI: 0.60–5.38, and for the case-crossover study: OR 1.62, 95% CI: 0.31–8.40.

## Discussion

The aim of the study was to assess whether there is an association between IDPs and VGS-IE among individuals at high risk in Sweden. The study could not confirm such an association exists, before or after the recommended cessation of AP for the prevention of VGS-IE in Sweden. Notably, AP for the prevention of IE is not routinely recommended in Sweden since October 2012,^12^ and amoxicillin prescriptions of among dentists decreased by 41% during the five following years; from 3,2 prescriptions per 1000 individuals per year in 2011 to 1,9 per 1000 individuals per year in 2017.^21^ Moreover, 95% of cases of VGS-IE among individuals at high risk were not temporally associated with IDPs, and may instead be attributed to daily bacteraemia caused by activities such as chewing or brushing. Following all potential individuals at high risk detectable in medical registers since 1964 that underwent numerous IDPs over a period of almost ten years, only 11 cases of VGS-IE were temporally associated with IDPs. 4 of these occurred after the 2012 decision to abandon the use of AP for the prevention of IE. Although low, this proportion is supported by findings in previous studies.^4^^,^^7^ Any extrapolation of the results to infer on the efficacy of AP in reducing VGS-IE among risk individuals depends on the assumption that Swedish dentists have adhered to the recommendation not to prescribe AP to individuals at high risk. In Sweden, AP is, in general, prescribed by the dentist, and the dentist is ultimately responsible for the decision to administer or not to administer AP before dental procedures.^22^ Antibiotic Prophylaxis (AP) is recommended for several procedures in dentistry, for example prevention of sepsis and osteonecrosis. In October 2012 cessation of AP for the prevention of IE was recommended, while recommendations on AP for other purposes remained.

For this reason, one would not expect as dramatic a fall in amoxicillin prescriptions as seen in England if Swedish dentists adhere to the changed recommendation as diligently as dentists in the UK. The 41% fall in AP prescriptions indicated by national data may therefore indicate close adherence to guidance.

Thornhill et al. found an association between in-hospital IDPs (extractions and surgical tooth removal) and IE in England.^18^ This finding may be due to selection bias rather than a causal effect. Microbiological data was not reported, and individuals at risk of infection (e.g., healthcare associated *S. aureus*-IE) often undergo in-hospital extractions and oral surgical procedures to address oral foci of infection. In-hospital IDPs are regularly conducted prior to thoracic surgery, radiation therapy and immunosuppression,^23^ and healthcare-related IE is often caused by *S. aureus*.^24^^,^^25^ In the study, 5.6% of IDPs were scaling procedures that were not associated with IE.

The current study was unable to replicate the findings of Thornhill et al., who found an association between IDPs and all cause IE in the USA, OR: 2.00 (95% CI: 1.59–2.52).^17^^,^^26^ They found strong associations between IE and dental extractions; (OR = 11.08, 95% CI: 7.34–16.74) and between IE and oral surgical procedures (OR = 50.77, 95% CI: 20.79–123.98) conducted during one month prior to IE.^17^ However, 89% of IDPs—160,999 sessions—were scaling procedures that were negatively associated with IE (OR = 0.46, 95% CI: 0.26–0.81).

There are several differences between the current study and the study by Thornhill et al. In the study by Thornhill et al., the proportion of cases caused by VGS was not measured. IDPs are believed to cause VGS-IE,^27^ and VGS-IE represented 9–17% of IE cases in the USA during the study period.^25^^,^^28^ Assuming the association that Thornhill et al. found between IDPs and IE was driven by VGS-IE, it should be several times higher between VGS-IE and IDPs, which is yet to be confirmed.^4^^,^^13^^,^^14^ The sample size of the current study is also larger than that of the study by Thornhill et al., and statistically it should have higher precision, as it is targeted at VGS-IE rather that all-cause IE. The sample size may therefore be large enough to provide support for an association between IDPs and VGS-IE. The positive associations found by Thornhill et al. were based 14,179 IDPs (11,483 extractions and 2696 sessions of oral surgery) among 36,773 individuals at high risk, while the current study was on 98,247 IDPs among 76,762 individuals at high risk.

The current study has several strengths. In-hospital procedures were not included, because in-hospital procedures are regionally funded by the counties rather than the SIA. Due to using the SRIE, the current study contained only possible and definite cases of VGS-IE according to the modified Duke criteria, and quality data on causative microorganism. This is important because 25–27% of IE-cases in the cohort are caused by VGS.^5^ Previous large studies have used patient registers to define IE,^4^^,^^17^ which may be challenging, mainly due to the low positive predictive value of several ICD-codes for IE,^29^ and because there are no specific codes to identify VGS-IE.^30^ Secondly, the current study design is not affected by recall bias or survivorship bias because it is based on registry data reported by health professionals rather than patient interviews.

There are limitations to the current study. Confounding by indication occurs if IE is caused by an infection with dental origin or gingival inflammation that leads to an IDP, rather than the IDP itself. However, such confounding would lead to overestimation of the association between IDPs and VGS-IE, and we detected no such association.

Measurement bias due to reverse causation occurs if IE is already present when the IDP is carried out. This may happen because diagnosis of IE is notoriously difficult, with unspecific symptoms. In Sweden, the national care program for IE states that the patient should undergo a dental examination during IE treatment, to detect and remove sources of infection in the oral cavity.^31^ This treatment could lead to IDPs being carried out approximately at the time of IE diagnosis. However, such confounding would also lead to overestimation of the association between IDPs and VGS-IE, and the current study detected no such association.

Selection bias can occur in case-control studies if controls differ substantially from cases regarding confounders such as comorbidities, dental health, and oral habits. In the current case-control study, educational attainment and comorbidities were measured and adjusted for in the analysis. The case-control design was complemented with a case-crossover design to account for measured and unmeasured confounders not varying over time. None of the studies detected an association between IDPs and VGS-IE.

Recommendations on AP have changed during the study period. If the frequency of VGS-IE is elevated following IDPs, and AP effectively reduces VGS-IE, the use of AP may have masked any association between July 2008 and October 2012. During this time, 2 g of amoxicillin per os was recommended 1 h before IDPs to individuals at high risk included in this study (patients with valve prosthesis, complicated heart valve disease, or previous endocarditis).^11^ Nevertheless, a masking presupposes a reduced number of cases from January 2008 to October 2012, yet most (64%) of the cases occurred during this period (Fig. 3).

The lack of a statistically significant association between IDPs and VGS-IE may also be due to a lack of power to detect an association. In terms of sample size, study design and evidence regarding any association between IDPs and VGS-IE, the study is most similar to the study by Tubiana et al.^4^ This French study included both a cohort design and a nested case-crossover on 103,463 IDPs among 138,876 individuals with prosthetic heart valves. AP was administered in 51% of IDPs and in four of the 14 cases temporally associated with IDPs. The study found a slight association between IDPs and VGS-IE (OR = 1.66, 95% CI: 1.05–2.63), but AP did not reduce this association.

The level of compliance to recommendations is unknown, although studies from USA and France (where AP for the prevention of IE is still recommended according to guidelines) indicate individuals at high risk receive AP prior to 30–50% of IDPs.^4^^,^^17^

The proportion of culture negative IE was 7% during the study period.^5^^,^^32^ However, as the proportion of culture negative did not increase after October 2012, there would have to be a substantial shift from other microorganisms to VGS-IE to introduce differential bias that could mask an increase in VGS-IE.^5^^,^^32^

In the absence of an RCT-study on the efficacy of AP in dentistry for the prevention of VGS-IE, evidence from countries where cessation has been recommended and AP prescriptions have dropped may provide guidance for expert committees tasked with producing recommendations and guidelines.

In conclusion, the study found no evidence that VGS-IE is associated with IDPs among individuals at high risk. The finding of a small (<5%) proportion of cases temporally associated with IDPs is similar to that of the previous large-scale study on IDPs and VGS-IE.

## Contributors

All authors confirm that they had full access to all the data in the study and accept responsibility for the decision to submit for publication.

Aron Naimi-Akbar determined the study design and verified the underlying data.

Niko Vähäsarja and Aron Naimi-Akbar contribute to the data curation.

Niko Vähäsarja, Aron Naimi-Akbar, Bodil Lund, Anders Ternhag, Margareta Hultin, Lars Olaison and Carina Krüger Weiner contributed equally to the literature search, figures, study design, data collection, data analysis, data interpretation, conceptualisation, formal analysis, funding acquisition, investigation, methodology, project administration, resources, software, supervision, validation, visualisation, writing of the original draft, and writing & editing of the reviewed draft.

## Data sharing statement

The study was conducted using national Swedish registries. The data that support the findings of this study are available from the SRIE, Statistics Sweden and the Swedish National Board of Health and Welfare but restrictions apply to the availability of these data, which were used under license for the current study, and so are not publicly available.

## Declaration of interests

The authors have no competing interests to declare.

## References

[1] Østergaard L., Valeur N., Ihlemann N. (2018). Incidence of infective endocarditis among patients considered at high risk. Eur Heart J.

[2] Steckelberg J.M., Wilson W.R. (1993). Risk factors for infective endocarditis. Infect Dis Clin North Am.

[3] Gersony W.M., Hayes C.J., Driscoll D.J. (1993). Bacterial endocarditis in patients with aortic stenosis, pulmonary stenosis, or ventricular septal defect. Circulation.

[4] Tubiana S., Blotière P.O., Hoen B. (2017). Dental procedures, antibiotic prophylaxis, and endocarditis among people with prosthetic heart valves: nationwide population based cohort and a case crossover study. BMJ.

[5] Vähäsarja N., Lund B., Ternhag A. (2022). Infective endocarditis among high-risk individuals - before and after the cessation of antibiotic prophylaxis in dentistry: a national cohort study. Clin Infect Dis.

[6] Quan T.P., Muller-Pebody B., Fawcett N. (2020). Investigation of the impact of the NICE guidelines regarding antibiotic prophylaxis during invasive dental procedures on the incidence of infective endocarditis in England: an electronic health records study. BMC Med.

[7] Thornhill M.H., Crum A., Rex S. (2022). Infective endocarditis following invasive dental procedures: IDEA case-crossover study. Health Technol Assess.

[8] Wilson W.R., Gewitz M., Lockhart P.B. (2021). Prevention of viridans group streptococcal infective endocarditis: a scientific statement from the American Heart Association. Circulation.

[9] Habib G., Lancellotti P., Antunes M.J. (2015). 2015 ESC guidelines for the management of infective endocarditis: the task force for the management of infective endocarditis of the European Society of Cardiology (ESC). Endorsed by: European Association for Cardio-Thoracic Surgery (EACTS), the European Association of Nuclear Medicine (EANM). Eur Heart J.

[10] Ljung R., Lundgren F., Appelquist M., Cederlund A. (2019). The Swedish dental health register - validation study of remaining and intact teeth. BMC Oral Health.

[11] von Elm E., Altman D.G., Egger M. (2014). The strengthening the reporting of observational studies in epidemiology (STROBE) statement: guidelines for reporting observational studies. Int J Surg.

[12] MPA (2012). https://lakemedelsverket.se/malgrupp/Halso---sjukvard/Behandlings--rekommendationer/Behandlingsrekommendation---listan/-Antibiotikaprofylax-i-tandvarden/.

[13] Lacassin F., Hoen B., Leport C. (1995). Procedures associated with infective endocarditis in adults. A case control study. Eur Heart J.

[14] Strom B.L., Abrutyn E., Berlin J.A. (1998). Dental and cardiac risk factors for infective endocarditis. A population-based, case-control study. Ann Intern Med.

[15] Porat Ben-Amy D., Littner M., Siegman-Igra Y. (2009). Are dental procedures an important risk factor for infective endocarditis? A case-crossover study. Eur J Clin Microbiol Infect Dis.

[16] Chen P.C., Tung Y.C., Wu P.W. (2015). Dental procedures and the risk of infective endocarditis. Medicine.

[17] Thornhill M.H., Gibson T.B., Yoon F. (2022). Antibiotic prophylaxis against infective endocarditis before invasive dental procedures. J Am Coll Cardiol.

[18] Thornhill M.H., Crum A., Campbell R. (2022). Temporal association between invasive procedures and infective endocarditis. Heart.

[19] Maclure M., Mittleman M.A. (2000). Should we use a case-crossover design?. Annu Rev Public Health.

[20] Arneborn P. (2017).

[21] Swedres-Svarm (2017).

[22] Läkemedelsverket MPA (2016). https://www.lakemedelsverket.se/sv/behandling-och-forskrivning/behandlingsrekommendationer/sok-behandlingsrekommendationer/antibiotikaprofylax-i-tandvarden---behandlingsrekommendation#hmainbody2.

[23] Smith M.M., Barbara D.W., Mauermann W.J., Viozzi C.F., Dearani J.A., Grim K.J. (2014). Morbidity and mortality associated with dental extraction before cardiac operation. Ann Thorac Surg.

[24] Que Y.A., Moreillon P. (2011). Infective endocarditis. Nat Rev Cardiol.

[25] Toyoda N., Chikwe J., Itagaki S., Gelijns A.C., Adams D.H., Egorova N.N. (2017). Trends in infective endocarditis in California and New York State, 1998-2013. JAMA.

[26] Thornhill M.H., Gibson T.B., Yoon F. (2023). Endocarditis, invasive dental procedures, and antibiotic prophylaxis efficacy in US Medicaid patients. Oral Dis.

[27] Habib G., Hoen B., Tornos P. (2009). Guidelines on the prevention, diagnosis, and treatment of infective endocarditis (new version 2009): the task force on the prevention, diagnosis, and treatment of infective endocarditis of the European Society of Cardiology (ESC). Endorsed by the European Society of Clinical Microbiology and Infectious Diseases (ESCMID) and the International Society of Chemotherapy (ISC) for Infection and Cancer. Eur Heart J.

[28] DeSimone D.C., Lahr B.D., Anavekar N.S. (2021). Temporal trends of infective endocarditis in Olmsted County, Minnesota, between 1970 and 2018: a population-based analysis. Open Forum Infect Dis.

[29] Fawcett N., Young B., Peto L. (2019). 'Caveat emptor': the cautionary tale of endocarditis and the potential pitfalls of clinical coding data-an electronic health records study. BMC Med.

[30] Dayer M.J., Jones S., Prendergast B., Baddour L.M., Lockhart P.B., Thornhill M.H. (2015). Incidence of infective endocarditis in England, 2000-13: a secular trend, interrupted time-series analysis. Lancet.

[31] Andreas Berge C.E., Ekspong L., Kurland S. (2021). https://infektion.net/wp-content/uploads/2021/11/vardprogram-infektios-endokardit-2021.pdf.

[32] Vahasarja N., Lund B., Ternhag A. (2020). Incidence of infective endocarditis caused by viridans group streptococci in Sweden – effect of cessation of antibiotic prophylaxis in dentistry for risk individuals. J Oral Microbiol.

